# Supplementary material for: Building a growing genomic repository for maternal and fetal health through the PING Consortium
Source: Pediatr Res. 2025 Jan 3;98(2):519–31. doi: 10.1038/s41390-024-03793-1 (PMC12222536; doi:10.1038/s41390-024-03793-1)
Supplement: Supplementary file 1 — Supplementary Material - The PING Genomic Data Repository [file 41390_2024_3793_MOESM1_ESM.pdf]

Supplementary Table 1. Sequencing Status of Samples Currently in the Consortium's Biorepository.

Country (Institution)	Total		Case		Control	
			Infants	Mothers	Infants	Mothers
**Brazil (CNH)**	DNA Sampled	545	277	232	28	28
	Sequenced	533	257	220	28	28
**Colombia (CNH and INS/CDC)**	DNA Sampled	1,672	321	103	946	282
	Sequenced	668	99	87	250	231
**Puerto Rico (CNH)**	DNA Sampled	59	19	20	9	9
	Sequenced	25	8	9	2	6

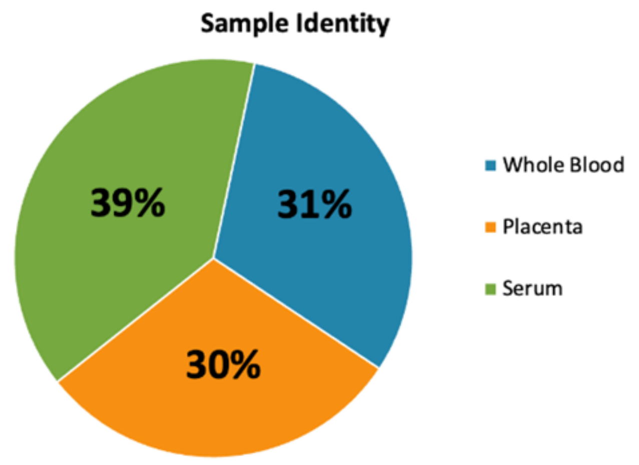

Supplementary Figure 1. Sample Identity of Biological Samples. The biorepository is comprised of a variety of sample identities. This includes blood (31%), placental tissue (30%) and serum (39%). All samples are undergoing DNA extraction for sequencing, analysis, and storage.
